# THE ROLE OF PSYCHOLOGICAL CAPITAL IN MODULATING CAREER ADAPTABILITY AND TURNOVER AMONG NURSING ASSISTANTS

**DOI:** 10.1093/geroni/igae098.3244

**Published:** 2024-12-31

**Authors:** Changxian Sun, Yurong Xing

**Affiliations:** Jiangsu Vocational Insititue of Commerce, Nanjing, Jiangsu, China (People’s Republic); Nanjing Medical University, Nanjing, Jiangsu, China (People’s Republic)

## Abstract

**Background:**

High turnover intention of nursing assistants was detrimental to the sustainability of long-term care. Career adaptability is an important determinant in reducing turnover intention, but little research has explored the mechanism from the perspective of psychological capital. The aim of this study was to analyze the association between career adaptability and turnover intention and to examine the mediating role of psychological capital between career adaptability and turnover intention among nursing assistants in mainland China.

**Methods:**

A cross-sectional online study was conducted among 276 nursing assistants from eight nursing homes in Nanjing, China. The participants’ career adaptability, psychological capital, and turnover intention were obtained. SPSS 26.0 and Amos 24.0 software were employed for statistical analysis.

**Results:**

Career adaptability was positively related to psychological capital and negatively linked to turnover intention (P < 0.01). Psychological capital played a fully mediating role (β = -0.085, P < 0.05) in the relationship between career adaptability and turnover intention, and the largest indirect effect was generated through the curiosity dimension. Conclusions: The management of long-term care facilities should focus on assessing the level of career adaptability of nursing assistants. The overall improvement of career adaptability and psychological capital is conducive in reducing turnover intention. Targeted interventions are recommended to improve career adaptability and reduce turnover intentions by increasing career curiosity. Online career adaptability programs can be developed for nursing assistant students to improve their psychological capital and facilitate career transitions.

